# Oseltamivir-Resistant Pandemic (H1N1) 2009 Virus, South Korea

**DOI:** 10.3201/eid1612.100600

**Published:** 2010-12

**Authors:** Hwajung Yi, Joo-Yeon Lee, Eun-Hye Hong, Mi-Seon Kim, Donghyok Kwon, Jang-Hoon Choi, Woo-Young Choi, Ki-Soon Kim, Jong-Koo Lee, Hee-Bok Oh, Chun Kang

**Affiliations:** Author affiliation: Korea Centers for Disease Control and Prevention, Seoul, South Korea

**Keywords:** Influenza, antiviral resistance, oseltamivir, neuraminidase, hemagglutinin, pandemic, H1N1, viruses, CME, dispatch

## MEDSCAPE CME

Medscape, LLC is pleased to provide online continuing medical education (CME) for this journal article, allowing clinicians the opportunity to earn CME credit. This activity has been planned and implemented in accordance with the Essential Areas and policies of the Accreditation Council for Continuing Medical Education through the joint sponsorship of Medscape, LLC and Emerging Infectious Diseases. Medscape, LLC is accredited by the ACCME to provide continuing medical education for physicians. Medscape, LLC designates this educational activity for a maximum of 0.25 *AMA PRA Category 1 Credits*™. Physicians should only claim credit commensurate with the extent of their participation in the activity. All other clinicians completing this activity will be issued a certificate of participation. To participate in this journal CME activity: (1) review the learning objectives and author disclosures; (2) study the education content; (3) take the post-test and/or complete the evaluation at www.medscapecme.com/journal/eid; (4) view/print certificate.

## Learning Objectives

Upon completion of this activity, participants will be able to:

- Describe the pattern and characteristics of resistance seen with antiviral agents in pandemic (H1N1) 2009 in South Korea.

## MEDSCAPE CME Editor

**Thomas J. Gryczan, MS, **Technical Writer/Editor, *Emerging Infectious Diseases. Disclosure: Thomas J. Gryczan, MS, has disclosed no relevant financial relationships*.

## MEDSCAPE CME AUTHOR

**Desiree Lie, MD, MSEd, **Clinical Professor; Director of Research and Faculty Development, Department of Family Medicine, University of California, Irvine at Orange. *Disclosure: Désirée Lie, MD, MSEd, has disclosed the following relevant financial relationship: served as a nonproduct speaker for “Topics in Health” for Merck Speaker Services.*

## AUTHORS

*Disclosures:* ***Hwajung Yi, PhD; Joo-Yeon Lee, PhD; Eun-Hye Hong, BS; Mi-Seon Kim, BS; Donghyok Kwon, MS; Jang-Hoon Choi, MS; Woo-Young Choi, PhD; Ki-Soon Kim, PhD; Jong-Koo Lee, MD; Hee-Bok Oh, PhD;*** *and* ***Chun Kang, MS*** *, have disclosed no relevant financial relationships.*
