# Supplementary material for: Oseltamivir-Resistant Pandemic (H1N1) 2009 Virus, South Korea
Source: Emerg Infect Dis. 2010 Dec;16(12):1938–42. doi: 10.3201/eid1612.100600 (PMC3294558; doi:10.3201/eid1612.100600)
Supplement: Related texts

# Brief report: Oseltamivir-Resistant Pandemic (H1N1) 2009 Virus, South Korea

**DOI:** 10.3201/eid1612.100600

**Authors:** Hwajung Yi, Joo-Yeon Lee, Eun-Hye Hong, Mi-Seon Kim, Donghyok Kwon, Jang-Hoon Choi, Woo-Young Choi, Ki-Soon Kim, Jong-Koo Lee, Hee-Bok Oh, Chun Kang

**Affiliations:** Author affiliation: Korea Centers for Disease Control and Prevention, Seoul, South Korea

## Abstract

To identify oseltamivir resistance, we analyzed neuraminidase H275Y mutations in samples from 10 patients infected with pandemic (H1N1) 2009 virus in South Korea who had influenza that was refractory to antiviral treatment with this drug. A neuraminidase I117M mutation that might influence oseltamivir susceptibility was detected in sequential specimens from 1 patient.

Since April 2009, pandemic (H1N1) 2009 has spread worldwide and caused the first influenza pandemic of the 21st century. Pandemic (H1N1) 2009 virus initially showed resistance to amantadine but susceptibility to oseltamivir (*1*). Thereafter, 285 cases of oseltamivir-resistant pandemic viral infection were reported worldwide on April 14, 2010 (*2*). However, information is limited about oseltamivir-resistant pandemic influenza in South Korea. Monitoring of community circulation of oseltamivir-resistant viruses has not yet detected any evidence of oseltamivir resistance in South Korea. To identify these viruses, we conducted specific surveillance of antiviral drug–resistant infection in patients whose illness did not resolve after antiviral treatment.

## The Study

The study was reviewed and approved by ethics committees of relevant institutions and hospitals. After patients provided informed consent, we obtained >150 clinical specimens from patients in various hospitals in South Korea. Respiratory specimens (>60% nasopharyngeal swab specimens) were obtained during October 2009–January 2010 from patients whose illness had been clinically refractory to antiviral treatment since October 2009.

Viral RNAs were isolated from specimens of 10 patients (Table 1) by using the QIAamp viral RNA Mini Kit (QIAGEN, Crawley, UK). PCR products of the neuraminidase (NA) and matrix 2 (M2) genes were generated by reverse transcription–PCR with primers for NA (forward: 5′-AAATTAACGGGCAATTCCTCTCT-3′; reverse: 5′-CCGAAAATCCCACTGCATATGTAT-3′) and M2 (forward: 5′-CTAGCTCCAGTGCTGGTCTGA-3′; reverse: 5′-CTCAGGCACTCCTTCCGTAGA-3′). DNA sequences of NA and M2 reverse transcription–PCR products were analyzed by using the Big-Dye Terminator Sequencing Reaction Kit and an ABI 3700 DNA analyzer (Applied Biosystems, Foster City, CA, USA). A total of 58 NA sequences and 52 M2 sequences were obtained and analyzed by using the sequence analysis tool in the Influenza Sequences and Epitopes Database for detecting oseltamivir-resistance mutations (*3*).

**Table 1:** Characteristics of 10 patients infected with oseltamivir-resistant pandemic (H1N1) 2009 virus, South Korea, 2009

Patient	Age, y/ sex	Antiviral treatment and date	Date of specimen collection	Type of clinical specimen	Underlying condition
A	5/M	Oseltamivir, 30 mg 2×/d (Oct 29–Nov 4); 60 mg 2×/d (Nov 5–9)	Nov 6	Nasopharyngeal swab	Mitochondrial cytopathic changes (bedridden)
B	46/M	Oseltamivir, 150 mg 2×/d (Nov 6–15); zanamivir, 10 mg 2×/d (Nov 16–26)	Nov 18	Nasopharyngeal swab	Leukemia
C	1/F	Oseltamivir, 30 mg 2×/d (Nov 16–21); 60 mg 2×/d (Nov 22–Dec 8)	Nov 21 and 22	Nasopharyngeal swab	Brain damage (fatal)
D	2/M	Oseltamivir, 90 mg 2×/d (Nov 26–30); 180 mg and amantadine, 65 mg 2×/d (Dec 2–7)	Nov 22, Dec 2	Nasal suction	None
E	3/F	Oseltamivir, 30 mg 2×/d (Dec 1–5 and 7–11)	Dec 1, 7, and 9	Oropharyngeal swab (Dec 1, 7); nasopharyngeal swab (Dec 9)	Asthma
F	3/F	Oseltamivir, 45 mg 2×/d (Dec 10–15); 75 mg 2×/d (Dec 16–20)	Dec 18	Oropharyngeal swab	Delayed development
G	1/F	Oseltamivir, 30 mg 2×/d (Dec 1–5); peramivir, 75 mg 1×/day (Dec 12–18)	Dec 1, 5, and 15	Nasal/oropharyngeal swab	Myelodysplasia (fatal)
H	63/M	Oseltamivir, 150 mg 2×/d (Dec 7–15); zanamivir, 10 mg 2×/d (Dec 16–20)	Dec 16	Oropharyngeal swab	Diabetes
I	58/M	Oseltamivir, 150 mg 2×/d (Dec 16–18); peramivir, 600 mg 1×/d, amantadine, 100 mg 2×/d a day, ribavirin, 300 mg 1×/d (Dec 19–25); zanamivir, 10 mg 2×/d (Dec 26–2010 Jan 1)	Dec 26	Nasopharyngeal swab	Cancer
J	60/M	Oseltamivir, 75 mg 2×/d (Nov 30–Dec 2)	Dec 1	Viral RNA	Diabetes, cardiac disorders (fatal)

Ten patients were detected who had oseltamivir-resistant pandemic (H1N1) 2009 virus with the H275Y substitution in viral NA (Table 2). Oseltamivir resistance was associated with oseltamivir treatment on the basis of H275Y changes from the oseltamivir-sensitive genotypes to oseltamivir-resistant genotypes of viral NA in consecutive samples from the same patient. Furthermore, a novel NA (I117M) substitution that may be associated with oseltamivir resistance (*4**,**5*) was detected in specimens from 1 patient (patient G) who had myelodysplasia and received oseltamivir and peramivir (Table 1, Table 2).

**Table 2:** Amino acid differences in HA and NA of viruses from 10 patients infected with oseltamivir-resistant pandemic (H1N1) 2009 virus, South Korean, 2009*

Strain or patient	Sample†	Amino acid sequences																		
		NA			M2		HA													
		275	117		31		22	56	83	116	119	120	128	134	155	197	203	249	283	321
Kor/01‡§		**H**	**I**		N		K	N	S	S	I	T	S	A	G	A	**S**	V	K	V
A‡	1	Y	I		N		K	N	S	S	I	T	S	A	G	**T**	T	V	K	V
B	1	Y	I		N		K	N	S	**N**	**I**	**I**	S	A	G	A	T	V	K	V
C‡	1	Y	I		N		K	N	S	S	I	T	S	A	G	A	T	V	K	V
	2	Y	I		N		K	N	S	S	I	T	S	A	G	A	T	V	K	V
D	1	**H**	I		N		K	N	S	S	I	T	**P**	A	G	A	T	**M**	K	V
	2	Y	I		N		K	N	S	S	I	T	**P**	A	G	A	T	**M**	K	V
E	1	**H**	I		N		K	N	S	S	I	T	S	A	G	A	T	**L**	K	V
	2	**H**	I		N		K	N	S	S	I	T	S	A	G	A	T	**L**	K	V
	3	Y	I		N		K	N	S	S	I	T	S	A	G	A	T	**L**	K	V
F	1	Y	I		N		K	N	S	S	I	T	S	**T**	G	A	T	V	K	V
G	1	**H**	**M**		N		**R**	N	S	S	I	T	**P**	A	G	A	T	V	K	V
	2	**H**	**M**		N		**R**	N	S	S	I	T	**P**	A	G	A	T	V	K	V
	3	Y	**M**		N		**R**	**S**	S	S	I	T	**P**	A	**E**	A	T	V	K	V
H	1	Y	I		N		K	N	S	S	**M**	T	S	A	G	A	T	V	K	V
I	1	Y	I		N		K	N	S	S	I	T	S	A	G	A	T	V	**E**	V
J¶	1	Y	I		–		–	–	–	–	–	–	–	–	–	–	–	–	–	–
Cal/07#		**H**	I		N		K	N	**P**	S	I	T	S	A	G	**T**	**S**	V	K	**I**
Cal/04**		**H**	I		N		K	N	**P**	S	I	T	S	A	G	A	**S**	V	K	**I**

*HA, hemagglutinin; NA, neuraminidase; M, matrix. Gene sequences for 16 clinical specimens from 10 patients were submitted to GenBank (accession nos. CY060453–CY060498). Amino acids that differ are shown in **boldface**. NA sequence numbering uses N1 nomenclature. †Matches the temporal order shown in the Figure. ‡Viral isolation and phenotyping of oseltamivir resistance were performed. §Pandemic A (H1N1) virus from South Korea (GenBank accession nos. GQ132185, GQ131025, and GQ131023). ¶Specimen from patient J was viral RNA. –, not available. #A/California/07/2009 virus (GenBank accession nos. GQ377078, FJ969537, and FJ969540). **A/California/04/2009 virus (GenBank accession nos. FJ969517, FJ969513, and GQ280797).

In addition, we cultured viral isolates from clinical specimens (patients A and C) and evaluated antiviral susceptibility by measuring the dose of oseltamivir and zanamivir required for 50% inhibition (IC_50_) of NA activity. One isolate of pandemic (H1N1) 2009 virus with an oseltamivir-sensitive genotype (H275 in its NA) was susceptible to oseltamivir (IC_50_ 1.18 nmol/L) and zanamivir (IC_50_ 0.42 nmol/L). Viral isolates from patients A and C with an oseltamivir-resistant genotype (Y275 in NA) were resistant only to oseltamivir (IC_50_ 713.2 nmol/L and 359.4 nmol/L, respectively). Susceptibility to zanamivir was not altered whether NA contained Y275 or H275 (IC_50_ 0.13 nmol/L and 0.78 nmol/L, respectively).

Patients with oseltamivir-resistant pandemic (H1N1) 2009 were treated during hospitalization with oseltamivir alone or with a combination of other antiviral drugs (Table 2). Active surveillance that evaluated spread of oseltamivir-resistant viral infections among hospital staff, family members, and other patients who had contacted with or cared for the patients showed no evidence of virus transmission in the hospitals.

## Conclusions

During pandemic (H1N1) 2009, oseltamivir-resistant viral infections were found mainly in immunocompromised patients who were treated with antiviral drugs and chemoprophylaxis (*6*). In our study, genetic characteristics of oseltamivir resistance in virus isolates from all 10 patients showed the H275Y substitution in NA, resulting in phenotypic resistance to oseltamivir but not to zanamivir. In addition, a novel NA mutation (I117M) was detected in virus from patient G (Table 1, Table 2). The NA 117 residue is near R118 and may have an effect on this residue, 1 of 3 arginine residues that bind the carboxylate region of the sialic acid substrate (*5**,**7*). This finding implies that changes at NA residue 117 may influence oseltamivir susceptibility of the virus. For example, the NA I117V mutation in avian influenza virus (H5N1) was reported to increase resistance to oseltamivir (*5**,**6*). The 117 residue is also highly conserved in all N1 typed viruses (*5*). These results indicate that the I117M substitution in NA of pandemic (H1N1) 2009 virus may influence oseltamivir resistance of this virus.

Multiple specimens were available from patients D, E, and G for analyzing antiviral resistance mutations in NA. Analysis of viruses from these patients showed that the H275Y mutation emerged in the later stages of viral infection, during oseltamivir treatment; infective viruses initially had an oseltamivir-sensitive genotype (H275) (Table 2; Figure).

**Figure:** Evolution of oseltamivir-sensitive H275 in pandemic (H1N1) 2009 virus to oseltamivir-resistant virus (mutation Y275) in the neuraminidase (NA) gene, South Korea. A) NA sequences of viruses from patient A, patient H, and strain A/Korea/01/2009 (H1N1) (Control) (Table 2). Viruses from these 2 patients had triplet TAC (encoding tyrosine in NA 275), indicating that these patients were infected with oseltamivir-resistant virus, in contrast to A/Korea/01/2009 (H1N1) of the oseltamivir-sensitive control showing CAC (encoding histidine) in NA 275. B) NA sequences from specimens obtained on different dates from patient E. This patient was initially infected with oseltamivir-sensitive virus with CAC in NA 275 (2009 Dec 1). Virus then showed mutation 823C (blue dominant peak) in the boxed CAC and a minor signal 823T (small red peak) in NA 275 (2009 Dec 7). In the third specimen (2009 Dec 9), only TAC in NA 275 was observed. C) Oseltamivir-sensitive virus infection (CAC in NA 275) was observed in the first 2 specimens from patient G (2009 Dec 1 and Dec 5). Mixed signals of dominant 823T (red peak) and minor 823C (blue peak) forms were seen in most recent specimen (2009 Dec 15), indicating a mixed infection with oseltamivir-resistant virus and oseltamivir-sensitive virus. Nucleotide coordinates (815 and 831) refer to NA gene sequence of pandemic (H1N1) 2009 virus. Amino acid residue 275 in the NA gene is encoded by 3 nt indicated in boxes.

A mixture of oseltamivir-sensitive (H275 in NA) and oseltamivir-resistant (Y275 in NA) viruses was observed in the second specimen from patient E and the third specimen from patient G (Figure). These specimens showed double signals (nucleotides T and C) for TAC and CAC encoding tyrosine (Y) and histidine (H), respectively. There are 2 possible explanations for mixtures of oseltamivir-resistant and oseltamivir-sensitive viral genotypes. The first explanation is reinfection with Y275 virus after a previous infection with H275 virus. The second explanation is an H275Y mutation caused by selective pressure from oseltamivir treatment. Our molecular epidemiologic data (Table 2) support the second explanation that selective pressure from oseltamivir treatment caused the H275Y substitution in NA because sequential viruses from the same patient had identical HA sequences in the absence of additional infection (*8*).

Multiple specimens from the same patient showed identical hemagglutinin sequences despite amino acid changes (H to Y) at position 275 of NA (hemagglutinin sequences of patients D, E, and G) (Table 2). This finding indicates that temporally sequential viruses from a patient were likely generated from the same viral progenitor without further viral infection. Conversely, our findings also support absence of epidemiologic links among current cases of oseltamivir resistance, given that viruses from 9 patients in this study had their own signature hemagglutinin amino acid sequences (Table 2).

Spreading and clustering of oseltamivir-resistant strains in the general population has not been observed in South Korea. However, in the present study, a comparatively high rate of emergence (19%) of oseltamivir-resistant viral infections was observed in unhealthy patients treated with antiviral drugs. We also detected a novel substitution (I117M) in NA, which might influence susceptibility to NA inhibitors. Our findings demonstrate that oseltamivir-resistant quasispecies of pandemic (H1N1) 2009 viruses can be generated. This suggestion indicates that continuous surveillance is required to evaluate emergence and circulation of drug-resistant pandemic (H1N1) 2009 viruses and possible reassortment with other viruses that have oseltamivir resistance.

## References

[1] Novel Swine-Origin Influenza A (H1N1) Virus Investigation Team, Dawood FS, Jain S, Finelli L, Shaw MW, Lindstrom S, et al. Emergence of a novel swine-origin influenza A (H1N1) virus in humans. N Engl J Med. 2009;360:2605–15. 10.1056/NEJMoa090381019423869

[2] World Health Organization. Weekly update on oseltamivir resistance to pandemic influenza A (H1N1) 2009 update 99, April [cited 2010 Aug 31]. http://www.who.int/csr/disease/swineflu/oseltamivirresistant2010416.pdf

[3] Yang IS, Lee JY, Lee JS, Mitchell WP, Oh HB, Kang C, Influenza sequence and epitope database. Nucleic Acids Res. 2009;37:D423–30. 10.1093/nar/gkn88119015128PMC2686482

[4] Govorkova EA, Ilyushina NA, McClaren JL, Naipospos TS, Douangngeun B, Webster RG. Susceptibility of highly pathogenic H5N1 influenza viruses to the neuraminidase inhibitor oseltamivir differs in vitro and in a mouse model. Antimicrob Agents Chemother. 2009;53:3088–96. 10.1128/AAC.01667-0819349520PMC2704685

[5] Hurt AC, Selleck P, Komadina N, Shaw R, Brown L, Barr IG. Susceptibility of highly pathogenic A (H5N1) avian influenza viruses to the neuraminidase inhibitors and adamantanes. Antiviral Res. 2007;73:228–31. 10.1016/j.antiviral.2006.10.00417112602

[6] Update on oseltamivir resistant pandemic A (H1N1) 2009 influenza virus: January 2010. Wkly Epidemiol Rec. 2009;85:37–40.20151493

[7] Russell RJ, Haire LF, Stevens DJ, Collins PJ, Lin YP, Blackburn GM, The structure of H5N1 avian influenza neuraminidase suggests new opportunities for drug design. Nature. 2006;443:45–9. 10.1038/nature0511416915235

[8] Gubareva LV, Novikov DV, Hayden FG. Assessment of hemagglutinin sequence heterogeneity during influenza virus transmission in families. J Infect Dis. 2002;186:1575–81. 10.1086/34537212447732

# Brief report: Oseltamivir-Resistant Pandemic (H1N1) 2009 Virus, South Korea

**DOI:** 10.3201/eid1612.100600

**Authors:** Hwajung Yi, Joo-Yeon Lee, Eun-Hye Hong, Mi-Seon Kim, Donghyok Kwon, Jang-Hoon Choi, Woo-Young Choi, Ki-Soon Kim, Jong-Koo Lee, Hee-Bok Oh, Chun Kang

**Affiliations:** Author affiliation: Korea Centers for Disease Control and Prevention, Seoul, South Korea

## Earning CME Credit

To obtain credit, you should first read the journal article. After reading the article, you should be able to answer the following, related, multiple-choice questions. To complete the questions and earn continuing medical education (CME) credit, please go to **www.medscapecme.com/journal/eid**. Credit cannot be obtained for tests completed on paper, although you may use the worksheet below to keep a record of your answers. You must be a registered user on Medscape.com. If you are not registered on Medscape.com, please click on the New Users: Free Registration link on the left hand side of the website to register. Only one answer is correct for each question. Once you successfully answer all post-test questions you will be able to view and/or print your certificate. For questions regarding the content of this activity, contact the accredited provider, CME@medscape.net. For technical assistance, contact CME@webmd.net. American Medical Association’s Physician’s Recognition Award (AMA PRA) credits are accepted in the US as evidence of participation in CME activities. For further information on this award, please refer to http://www.ama-assn.org/ama/pub/category/2922.html. The AMA has determined that physicians not licensed in the US who participate in this CME activity are eligible for *AMA PRA Category 1 Credits*™. Through agreements that the AMA has made with agencies in some countries, AMA PRA credit is acceptable as evidence of participation in CME activities. If you are not licensed in the US and want to obtain an AMA PRA CME credit, please complete the questions online, print the certificate and present it to your national medical association.

## Article Title: Oseltamivir-Resistant Pandemic (H1N1) 2009 Virus, South Korea

## CME Questions

**1. Which of the following best describes the pattern of oseltamivir resistance seen with pandemic (H1N1) 2009 virus in South Korea?**A. Resistance was seen synchronous with amantadine resistanceB. Resistance was seen in the communityC. Resistance was seen in hospitalized patientsD. Resistance was seen only in immunosuppressed patients**2. A 45-year-old man from South Korea with pandemic (H1N1) 2009 infection is found to have resistance to oseltamivir. Which of the following is the most likely outcome of this resistance?**A. Increased transmission of virus to staff and familyB. Higher risk for resistance in the communityC. Increased length of hospital stayD. None of the above

## Activity Evaluation

**Table:** 

1. The activity supported the learning objectives.				
Strongly Disagree				Strongly Agree
1	2	3	4	5
2. The material was organized clearly for learning to occur.				
Strongly Disagree				Strongly Agree
1	2	3	4	5
3. The content learned from this activity will impact my practice.				
Strongly Disagree				Strongly Agree
1	2	3	4	5
4. The activity was presented objectively and free of commercial bias.				
Strongly Disagree				Strongly Agree
1	2	3	4	5
